# Toward target 2035: EUbOPEN - a public–private partnership to enable & unlock biology in the open

**DOI:** 10.1039/d4md00735b

**Published:** 2024-11-29

**Authors:** Claudia Tredup, Suzanne Ackloo, Hartmut Beck, Peter J. Brown, Alex N. Bullock, Alessio Ciulli, Ivan Dikic, Kristina Edfeldt, Aled M. Edwards, Jonathan M. Elkins, Henner F. Farin, Edward A. Fon, Matthias Gstaiger, Judith Günther, Anna-Lena Gustavsson, Sandra Häberle, Laura Isigkeit, Kilian V. M. Huber, Andras Kotschy, Oliver Krämer, Andrew R. Leach, Brian D. Marsden, Hisanori Matsui, Daniel Merk, Florian Montel, Monique P. C. Mulder, Susanne Müller, Dafydd R. Owen, Ewgenij Proschak, Sandra Röhm, Alexandra Stolz, Michael Sundström, Frank von Delft, Timothy M. Willson, Cheryl H. Arrowsmith, Stefan Knapp

**Affiliations:** a Institute of Pharmaceutical Chemistry, Goethe University Frankfurt Frankfurt 60438 Germany knapp@pharmchem.uni-frankfurt.de; b Structural Genomics Consortium, BMLS, Goethe University Frankfurt Frankfurt 60438 Germany; c Structural Genomics Consortium, University of Toronto - St George Campus 101 College Street, MaRS Center South Tower 7th Floor Toronto Canada; d Drug Discovery Sciences, Research & Development, Pharmaceuticals, Bayer AG Wuppertal Nordrhein-Westfalen Germany; e Structural Genomics Consortium, University of North Carolina at Chapel Hill Campus Box 7356, 120 Mason Farm Road, GMB 1070 Chapel Hill North Carolina USA; f Centre for Medicines Discovery, University of Oxford NDM Research Building, Roosevelt Drive Oxford Oxfordshire UK; g Centre for Targeted Protein Degradation, University of Dundee, School of Life Sciences 1 James Lindsay Place DD1 5JJ Dundee UK; h Institute of Biochemistry II, Goethe University Frankfurt, Medical Faculty Frankfurt am Main Germany; i Buchmann Institute for Molecular Lifesciences, Goethe University Frankfurt Frankfurt am Main Germany; j Structural Genomics Consortium, Department of Medicine, Karolinska University Hospital and Karolinska Institutet Stockholm Sweden; k Georg-Speyer-Haus, Institute for Tumor Biology and Experimental Therapy Frankfurt am Main Hessen Germany; l Department of Neurology and Neurosurgery, Montreal Neurological Institute-Hospital (The Neuro), McGill University Montreal Canada; m Department of Biology, Institute of Molecular Systems Biology ETH Zürich Zurich ZH Switzerland; n Drug Discovery Sciences, Bayer AG Berlin Germany; o Chemical Biology Consortium Sweden, Department of Medical Biochemistry & Biophysics, Karolinska Institute Stockholm Sweden; p Servier Research Institute of Medicinal Chemistry Budapest Hungary; q Discovery Research Coordination, Boehringer Ingelheim International GmbH Binger Straße 173 55216 Ingelheim am Rhein Germany; r European Bioinformatics Institute (EMBL-EBI), Wellcome Genome Campus Hinxton Cambridge UK; s Neuroscience Drug Discovery Unit, Takeda Pharmaceutical Company Limited Fujisawa Kanagawa Japan; t Ludwig-Maximilians-Universitat Munchen Munchen Germany; u Discovery Research Coordination, Boehringer Ingelheim Pharma GmbH & Co. KG Birkendorfer Straße 65 88397 Biberach an der Riss Germany; v Department of Cell and Chemical Biology, Leiden University Medical Center Leiden The Netherlands; w Pfizer Medicine Design Cambridge MA 02139 USA; x Diamond Light Source, Harwell Science and Innovation Campus Didcot OX11 0DE UK; y Princess Margaret Cancer Centre Toronto Ontario M5G 1L7 Canada; z Fraunhofer Institute for Translational Medicine and Pharmacology ITMP Theodor-Stern-Kai 7 60596 Frankfurt am Main Germany

## Abstract

Target 2035 is a global initiative that seeks to identify a pharmacological modulator of most human proteins by the year 2035. As part of an ongoing series of annual updates of this initiative, we summarise here the efforts of the EUbOPEN project whose objectives and results are making a strong contribution to the goals of Target 2035. EUbOPEN is a public–private partnership with four pillars of activity: (1) chemogenomic library collections, (2) chemical probe discovery and technology development for hit-to-lead chemistry, (3) profiling of bioactive compounds in patient-derived disease assays, and (4) collection, storage and dissemination of project-wide data and reagents. The substantial outputs of this programme include a chemogenomic compound library covering one third of the druggable proteome, as well as 100 chemical probes, both profiled in patient derived assays, as well as hundreds of data sets deposited in existing public data repositories and a project-specific data resource for exploring EUbOPEN outputs.

## Abstract 2

EUbOPEN is a public–private partnership focused on four areas: chemogenomic library collection, chemical probe discovery and technology development, compound profiling in patient-derived assays, and data and reagents collection, storage and sharing.

## Graphical abstract

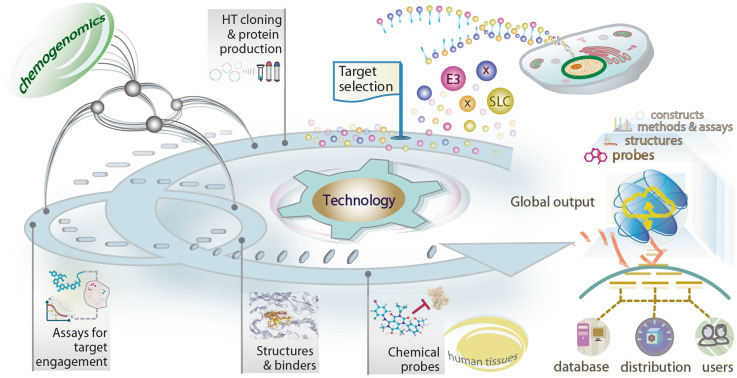

## Introduction

Current small-molecule drug development focuses on a few well-established target families that define the explored druggable proteome. Although the number of target families has increased significantly over the past few decades, many proteins within established and yet to be discovered target families remain unexplored. Sequencing effort has identified many disease-associated mutations that provide a compelling rationale for targeting these proteins for the development of new therapeutics. However, the druggability of most of these proteins has not yet been demonstrated by the development of selective and potent small molecules. Such modulators, also called chemical probes, are powerful tools for the validation of new targeting strategies.^1^ Yet, the combined effort of the private sector and academic drug development community has only developed a few hundred high quality chemical probes to date. The current long development timelines preclude the development of chemical probes for every druggable protein in the near future. However, potent inhibitors or chemical activators with narrow but not exclusive target selectivity, so-called chemogenomic (CG) compounds, are a feasible and extremely useful interim solution until more selective molecules are discovered. CG tools are powerful reagents when several of these small molecules with diverse off-target activity profiles are combined into collections of compounds that allow target deconvolution based on selectivity patterns.^2^ The combined collection of high-quality chemical probes and well annotated CG compound sets covers now a much larger target space. The selection and characterization of CG sets for the druggable protein families is one of the major goals of the EUbOPEN consortium.

Recently, new modalities such as molecular glues, PROTACs (PROteolysis TArgeting Chimeras) and other proximity inducing small molecules such as molecular glues have also emerged as new chemical modulators with unique properties.^3–5^ This has led to a large expansion of the druggable proteome and new opportunities for drug development. However, only a few of the large family of E3 ligases have been successfully exploited so far, limiting our ability to develop the next generation of chemical degraders. The development of new E3 ligase ligands and the identification of linker attachment points, known as E3 handles, for degrader design has also been a major goal of EUbOPEN, and the first new E3 ligands have now been published by the consortium.^6–8^

Target 2035 is an international open science initiative that aims to generate chemical or biological modulators for nearly all human proteins by 2035.^1,9–12^ Initially defined by scientists from academia and the pharmaceutical industry and driven by the Structural Genomics Consortium (SGC), this initiative has grown into a global effort with the goal of making chemical and biological tools and data freely available to the research community. Importantly, the developed tools are peer reviewed, to promote confidence that they are fit for purpose in studying the target of interest.^1,13,14^ A major contributor to these efforts is the European Union Innovative Medicines Initiative project “Enabling and Unlocking Biology in the OPEN”, or ‘EUbOPEN’ for short. Here, we provide an overview and summary of recent achievements of EUbOPEN, and comment on how they enhance Target 2035 activities.

## EUbOPEN as a major contributor to target 2035

EUbOPEN is a public–private partnership with ambitious goals to create, distribute and annotate the largest openly available set of high-quality chemical modulators for human proteins https://www.eubopen.org/. EUbOPEN consists of 22 partners from academia and the pharmaceutical industry, working together on projects that include novel chemical probes for emerging target areas such as solute carriers (SLCs), E3-ubiquitin ligases (E3s) and other understudied target families, as well as a chemogenomic (CG) library covering one third of the druggable genome. All compounds in the collections are comprehensively characterised for their potency, selectivity, and cellular activity. The compounds are annotated with a suite of biochemical and cell-based assays, including those derived from primary patient cells. Diseases of particular focus for the development of primary cell assays include inflammatory bowel disease, cancer and neurodegeneration. Finally, the development of new technologies to significantly shorten the hit identification and the hit-to-lead optimisation processes, aim to provide the foundation for future efforts toward Target 2035.

## Developing, validating and distributing peer reviewed chemical probes

Chemical probes represent the gold standard amongst chemical tools. They are highly characterised, potent and selective, cell-active small molecules that modulate the function of a protein.^1,15,16^ Commonly, these molecules are inhibitors or degraders, but for some target families, such as G-protein coupled receptors (GPCRs) other forms of modulations, such as agonist or antagonist activities, exist. Strict criteria for chemical probes have been agreed upon by the EUbOPEN consortium members. These criteria include potency measured in *in vitro* assays of less than 100 nM, a selectivity of at least 30 fold over related proteins, evidence of target engagement in cells at less than 1 μM or 10 μM for shallow protein–protein interaction targets, and a reasonable cellular toxicity window (unless cell death is target mediated). EUbOPEN aims to deliver 50 new, collaboratively developed chemical probes within the 5-year programme with a specific focus on E3 ligases and SLCs. EUbOPEN scientists have ongoing collaborations with many academic researchers outside of the consortium as well as five pharmaceutical companies that are members of the European Federation of Pharmaceutical Industries and Associations (EFPIA). These collaborations are conducted in a pre-competitive manner with EUbOPEN scientists from multiple academic institutions. All chemical probes developed by the EUbOPEN consortium are peer reviewed by an external committee and released with a structurally similar inactive negative control compound.

The focus of EUbOPEN on novel challenging target classes, in particular ubiquitin E3 ligases, given their roles as attractive targets in their own right, and as the enzymes hijacked/co-opted by degrader molecules such as molecular glues and PROTACs, has pushed the field to revisit criteria and to include new modalities *e.g.* covalent binders and PROTACs, and E3 ligase handles.^16^ A recent output from EUbOPEN illustrates a fitting case study and a compelling example and template for developing chemical probes that meet these evolving qualifying utility criteria. Small-molecule covalent inhibitors of the Cul5-RING ubiquitin E3 ligase substrate receptor subunit SOCS2 were designed by Ramachandran *et al.*^17^ to target the hard-to-drug SH2 domain and shown to block substrate recruitment *in vitro* and within cells. Starting from phosho-tyrosine as an anchor bound fragment, crystallographic structure-based design guided the optimisation of compounds with high ligand efficiency. The qualified E3 ligase handle/probe molecule featured effective covalent modification of a specific cysteine residue on the SOCS2 SH2 domain binding site, and a pro-drug strategy to mask the phosphate group and aid cell permeability.^17^

EUbOPEN aims to collate and make openly available additional 50 high-quality chemical probes from the community. In this unique project, the Donated Chemical Probes (DCP) project, chemical probes developed by academics and/or industry are peer-reviewed by two independent committees on the chemical probe criteria and made available to researcher worldwide without any restrictions on use.^15,18^ So far, EUbOPEN is on track to generate or collect in total 100 high quality chemical probes from the community by May 2025. All compounds will be freely and openly available and can be requested *via*https://www.eubopen.org/chemical-probes. An information sheet with key data and recommendations for their use in cellular assays is provided to avoid or minimise off-target effects when using these compounds.^19^ To date, EUbOPEN distributed more than 6000 samples of chemical probes and controls to researchers around the world without restrictions. This initiative aims to accelerate target validation and serve as a foundation for drug discovery. By concentrating on targets where the link to diseases is poorly understood, new applications for small molecule ligands could be uncovered.

## Chemogenomic compound collections covering one third of the druggable proteome

The generation of chemical probes can be both costly and challenging, particularly when it comes to optimising a compound for high selectivity within the target family. Achieving this level of selectivity is not always feasible, which limits the potential to create chemical probes for a wide range of targets. To overcome these limitations and address a significant portion of the druggable genome, a chemogenomics strategy has been applied. Chemogenomic compounds, in contrast to chemical probes, may bind to multiple targets but are still valuable due to their well-characterized target profiles. By leveraging chemogenomic compound sets, researchers can systematically explore interactions between small molecules and a broad spectrum of biological targets, providing insights into druggable pathways and enhancing the efficiency of drug discovery. In addition to data from the literature, EUbOPEN has set up several selectivity panels for different target families to further annotate these compounds. By using a set of these well-characterised compounds with overlapping target profiles, the target responsible for a specific phenotype can be identified. Family-specific criteria have been defined with the help of an external expert committee from different target areas, taking into account the availability of well-characterised compounds and screening possibilities, ligandability of different targets and the possibility to collate more than one chemotype per target. The criteria can be viewed at https://www.eubopen.org/chemogenomics/chemogenomics-criteria.

Hundreds of thousands of bioactive compounds generated by medicinal chemistry efforts in the industrial and academic sectors, together with the availability of public compound/bioactivity databases, facilitated the assembly of highly annotated CG libraries for large target families. When EUbOPEN was launched in 2020, prominent public repositories contained a total of 566 735 compounds with a target-associated bioactivity ≤10 μM covering 2899 human target proteins as CG compound candidates.^20^ Kinase inhibitors and G-protein coupled receptor (GPCR) ligands dominate the annotated compounds, as these have long been the focus of medicinal chemistry, but several other target families are sufficiently represented for the development of high-quality CG sets. Moreover, at the time of writing, 7818 candidate compounds are commercially available, enabling the assembly of sustainable libraries with broad accessibility for the scientific community. Benefitting from these valuable resources, we set out to assemble CG sets for several large target families. Based upon data from leading public repositories and an in-house curated bioactivity database,^20^ we identified CG compound candidates meeting our stringent family-specific quality criteria. The candidates underwent rigorous chemical and biological quality control, were validated for their intended activities and profiled for selectivity before potential inclusion in a CG set. Despite considerable attrition due to non-reproducibility of published bioactivity values, we were able to identify up to five chemically diverse CG compounds per target for orthogonality.^21^

Efforts to develop a dedicated kinase CG set have faced a high attrition rate of 30%, mainly due to insufficient potency and selectivity of kinase inhibitors, despite promising published profiles. All compounds are additionally characterised in biochemical and/or cellular assays and we have developed a gateway https://gateway.eubopen.org to make the compound characterisation data available to the public.^21–25^ This comprehensive effort ensures that the data is accessible for further research and development, supporting the scientific community in their endeavors to understand and target various biological pathways.

## Patient-derived disease tissue assays for target discovery and validation

Translational medical studies rely on data from disease relevant models. In EUbOPEN, we use our high-quality tool compounds to discover novel target-disease associations. Our specific aims are to develop models and assays based on samples from patients and control subjects (*e.g.* blood, tissue) and test the compounds in unbiased studies. We focus on inflammatory bowel diseases (IBD), colorectal cancer (CRC), liver fibrosis (LF) and multiple sclerosis (MS). In these areas, we developed and validated several patient-derived cell assays (PCAs) https://www.eubopen.org/tissue-assays, cooperating closely with hospitals, clinicians, and patients.

We have established patient-derived organoids (PDOs) from healthy and CRC tissues to study tumour-specific responses. Screening of chemical probes revealed patient-specific tumour vulnerabilities.^18^ Viability data from other cell systems allows for the identification of compounds with a favourable toxicity profile. PDOs were further co-cultured with matched fibroblasts to mimic native growth conditions. Subsequent co-cultures from 29 patients allowed us to study therapy resistance in the tumour microenvironment and the identification of compound combinations to overcome resistance.^26^ Furthermore, our focus is on the identification of chemical probes that can restore compromised intestinal epithelial barrier, promoting mucosal healing. Thus, we have established an automated screening platform using gastrointestinal PDOs co-cultured with matched fibroblasts and are testing the effects of chemical probes. Other developments include gut biomimetic scaffolds for controlled organoid growth^27^ and identification of pathways that uncouple intestinal regeneration from tumorigenesis. In addition, spheroid models for metabolic dysfunction-associated steatohepatitis (MASH) from patient-derived cells have been established and characterised, which showed that they recapitulate interactions between hepatocytes and immune cells and closely resembles the disease tissue using multi-dimensional omics.^28^ The system has been optimised for compound screening with phenotypic and functional readouts, *e.g.* inflammation and fibrogenesis. We have identified novel potential intervention points for therapy, including a novel GPCR-ion channel axis and other signalling networks controlling human hepatocyte regeneration.^29^

The important role of B cells and epigenetics in multiple sclerosis^30^ prompted us to establish a B cell assay using primary patient cells to test and measure effects of chemical probes and chemogenomic compounds on *e.g.* pro-inflammatory cytokines. Preliminary results have revealed interesting target-disease associations, which are now undergoing validation studies. In summary, we have established validated PCAs in four main disease areas and demonstrated the value of EUbOPEN chemogenomic libraries and chemical probes for the identification of new potential disease targets and biomarkers for further studies.

## Implementing FAIR principles in the EUbOPEN data management strategy

Publication of high quality, open datasets is also a core deliverable of EUbOPEN. Primarily this consists of data and metadata describing small molecules as part of chemogenomic or chemical tool collections and contains a broad range of data types derived from biophysical, biochemical and cellular assays. Additionally, data from structural biology, proteomics and biological probe generation are also made available.

Our experience has been that it is critical to capture data and metadata from day one in a well-defined and accessible manner to ensure close adherence to FAIR (findable, accessible, interoperable, and re-usable) principles. Within EUbOPEN, we have leveraged our data management approaches from the SGC and Oxford's Centre for Medicines Discovery, employing a laboratory information management system (LIMS), Scarab, from MolSoft LLC. This platform, which we call EUbScarab, is able to capture unstructured data, such as lab notebooks, as well as structured and workflow data. Importantly, Scarab can be adapted rapidly to accommodate new data types without needing any changes in its codebase. The use of EUbScarab has meant that the deposition path of chemical biology data into both ChEMBL and BioImage Archive has been relatively frictionless, as we have been able to adapt our data capture approaches to map tightly with the existing ChEMBL database schema, for example. Nevertheless, close collaboration with colleagues at the EMBL's European Bioinformatics Institute (EBI) has resulted in fruitful solutions to provide new ways of capturing and presenting EUbOPEN data to the community, ensuring long-term sustainability and adherence to FAIR principles.

EUbOPEN datasets are stored in the public resource most relevant to the data type so, for example, pharmacology data from bioassays is stored in ChEMBL and protein structures are stored in the wwPDB. The EUbOPEN Gateway is a bespoke web interface and supporting infrastructure that provides the global user community with facile access to EUbOPEN data. The gateway is designed to enable users who are not informatics experts to easily answer common questions and use-cases. These range from the relatively straightforward, such as “what chemical probes are available for my target?” and “what pharmacology data is available for this chemogenomic set compound?” to more complex queries such as “I have a number of active compounds from screening the chemogenomic set in my phenotypic assay: what target(s) are likely to be responsible?”

**Fig. 1:**
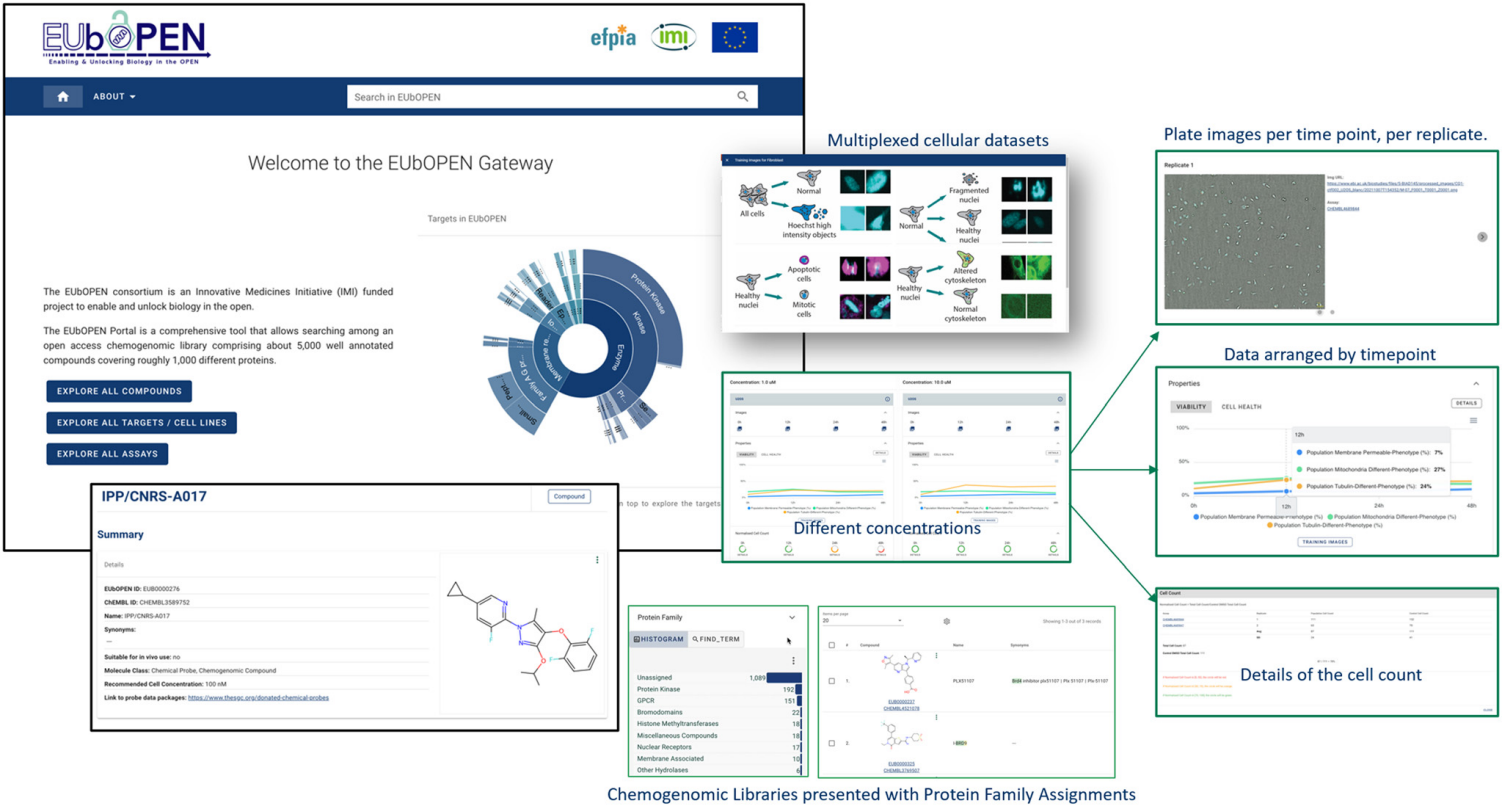
Screenshots from the EUbOPEN Gateway.

Various screenshots from the currently released version of the gateway can be seen in Fig. 1. Functions are provided to allow users to search, filter and visualise results across molecules, targets and assays. Of particular note is the development of capabilities to display complex multiplexed data, for example from cell-health assays. This example also illustrates how different data types are stored in the most relevant resource (here, pharmacology data in ChEMBL and imaging data in the BioImage Archive). The gateway moreover provides links to the underlying core databases to enable more detailed access, interrogation and to download the data for *in silico* applications.

To date, >600 000 activity data points from >14 000 assays covering >1500 targets have been deposited into ChEMBL. The EUbOPEN datasets constitute a highly valuable, high-quality, resource for chemical biology and drug discovery that will continue to evolve and grow to the end of the project and beyond. We anticipate that it will prove to be invaluable for advanced machine learning methods in the chemical biology space.

## Technology development for accelerating hit-to-lead chemistry

While chemical probes have proven to be powerful reagents,^1^ their discovery and development remains an ongoing challenge in medicinal chemistry and chemical biology. As in traditional drug discovery programmes, the process starts with the identification of bioactive small molecules, followed by iterative cycles of medicinal chemistry for lead generation. Additionally, characterising their selectivity profile is crucial to ensuring high-quality chemical probes.^31^ This laborious, time, and cost-intensive workflow emphasises the significance of automation and new technologies in expediting and enhancing the generation and characterisation of chemical probes, a focal point of the EUbOPEN project.

Computer aided drug design (CADD), fragment-based drug discovery (FBDD)^32^ and DNA-encoded library (DEL) technology^33^ have emerged as powerful hit-finding approaches, enabling the exploration of challenging targets and broadening the accessible boundaries of chemical space. Despite these advancements, challenges persist. While automation is widely used across various stages of drug discovery workflows,^34^ integration across adjacent stages remains uncommon. Moreover, the reliance on a limited reaction toolkit dominates molecular discovery, resulting in an uneven exploration of chemical space within drug discovery programmes.^35^

Initiatives within EUbOPEN focus on enhancing hit-to-lead timelines by expanding reaction toolkits and fragment sets for plate-based chemistry. Employing a structure-blind, function-driven approach, arrays of reactions are conducted *via* plate-based chemistry, with crude products screened for biological function, facilitating activity-directed discovery.^36^ Here, only bioactive products are purified for structure elucidation. Unlike conventional workflows favouring specific designed molecules, this approach allows exploration of chemistry with multiple regiochemical outcomes and functionalisation of different heteroatoms (*e.g.* metal–carbenoid chemistry).^37–39^

Another successful development involves methods for progressing hits from XChem crystallographic fragment screens. In an early proof-of-concept experiment based on fragment hits against the second bromodomain of PHIP (PHIP(2)),^40^ a diverse 2000-compound follow-up library was synthesised by low-cost robotics, and all products were tested as crude reaction mixtures by crystallography, as a second round XChem experiment. This effort yielded micromolar hits in both biochemical and biophysical assays,^41^ providing actionable structure–activity relationship (SAR) data from the crystal structures.^42^ This experiment worked because the crystallised protein can extract (purify) the active species from solution, and crystallography is sensitive enough to observe it. The result forms the basic premise underlying a new platform, Fast-forward fragments (FFF), which combines an experimental workflow with a new algorithmic approach for computing fragment merges, the fragment knitwork.^43^

Finally, as covalent libraries are highly valuable in biology and drug discovery, a platform for acoustic high-throughput automated synthesis^44^ facilitates rapid generation of diverse covalent screening libraries and parallel screening without purification.^45^ This method streamlines the synthesis of focused screening libraries, reducing the need for extensive medicinal chemistry efforts typically associated with fragment based drug discovery programmes.

Integrating automation with algorithm-assisted design enhances efficiency in drug discovery by optimizing reaction conditions, predicting molecular properties, and prioritizing compounds for further investigation.^46,47^ This integration also holds promise in addressing probe selectivity by enabling high-throughput screening against diverse targets and facilitating the prediction of selectivity profiles based on molecular properties and structural features. Combining these approaches with other EUbOPEN initiatives towards detailed probe selectivity profiles ensures the reliability and specificity of these reagents. Family-wide cellular screening platforms,^48,49^ as well as new mass spectrometry based technologies^50^ help streamline the process of probe development.^51^ Overall, this integrated approach accelerates the discovery of reliable and selective chemical tools for biological system interrogation.

## Outlook

By the end of the EUbOPEN project in May 2025, this initiative will have contributed more than 100 novel chemical probes and collected chemogenomic modulators covering 1000 targets. For each chemical probe, characterisation data will be available on the EUbOPEN Gateway as well as in the ChEMBL database enabling both human readability and machine learning. The current progress of this ambitious project indicates that these milestones are within reach, providing the chemical biology community with powerful tools for the exploitation of novel disease mechanisms.

Despite this major contribution of EUbOPEN to the exploration of the druggable proteome, much remains to be done. Many targets in established or previously unknown target families remain unexplored. In the future, more efficient and cost effective hit-finding strategies and advances in machine learning and AI technologies are expected to play pivotal roles in this area in the future. As these technologies rely on robust data for algorithm refinement and optimisation, the EUbOPEN Gateway will provide a crucial resource for machine learning technologies. In summary, while EUbOPEN will significantly enhance the chemical biology toolkit by 2025, continued innovation and investment will be critical for advancing drug discovery and understanding complex biological systems in the years to come.

## Disclaimer

This communication reflects the views of the authors and neither IMI nor the European Union, EFPIA or any Associated Partners are liable for any use that may be made of the information contained herein.

## Data availability

No primary research results, software or code have been included and no new data were generated or analysed as part of this review.

## Conflicts of interest

There are no conflicts to declare.
